# Risk factors and mortality for elderly patients with bloodstream infection of carbapenem resistance *Klebsiella pneumoniae*: a 10-year longitudinal study

**DOI:** 10.1186/s12877-022-03275-1

**Published:** 2022-07-13

**Authors:** Yili Chen, Yao Chen, Pingjuan Liu, Penghao Guo, Zhongwen Wu, Yaqin Peng, Jiankai Deng, Yannan Kong, Yingpeng Cui, Kang Liao, Bin Huang

**Affiliations:** 1 Department of Laboratory Medicine, The First Affiliated Hospital of Sun Yat-Sen University, Guangzhou, Guangdong China; 2 Department of Laboratory Medicine, the Nanhai’s Fifth People’s Hospital of Foshan, Guangdong, China

**Keywords:** Carbapenem-resistant K. pneumoniae, Bloodstream infection, Risk factor, Mortality, Elderly patients

## Abstract

**Background:**

Bloodstream infection (BSI) caused by carbapenem resistant *Klebsiella pneumoniae* (CRKP), especially in elderly patients, results in higher morbidity and mortality. The purpose of this study was to assess risk factors associated with CRKP BSI and short-term mortality among elderly patients in China.

**Methods:**

In this retrospective cohort study, we enrolled 252 inpatients aged ≥ 65 years with BSI caused by KP from January 2011 to December 2020 in China. Data regarding demographic, microbiological characteristics, and clinical outcome were collected.

**Result:**

Among the 252 BSI patients, there were 29 patients (11.5%) caused by CRKP and 223 patients (88.5%) by carbapenem-susceptible KP (CSKP). The overall 28-day mortality rate of elderly patients with a KP BSI episode was 10.7% (27/252), of which CRKP BSI patients (14 / 29, 48.3%) were significantly higher than CSKP patients (13 / 223, 5.83%) (*P* < 0.001). Hypertension (OR: 13.789, [95% CI: 3.883–48.969], *P* < 0.001), exposure to carbapenems (OR: 8.073, [95% CI: 2.066–31.537], *P* = 0.003), and ICU stay (OR: 11.180, [95% CI: 2.663–46.933], *P* = 0.001) were found to be associated with the development of CRKP BSI in elderly patients. A multivariate analysis showed that isolation of CRKP (OR 2.881, 95% CI 1.228–6.756, *P* = 0.015) and KP isolated in ICU (OR 11.731, 95% CI 4.226–32.563, *P* < 0.001) were independent risk factors for 28-day mortality of KP BSI.

**Conclusion:**

In elderly patients, hypertension, exposure to carbapenems and ICU stay were associated with the development of CRKP BSI. Active screening of CRKP for the high-risk populations, especially elderly patients, is significant for early detection and successful management of CRKP infection.

## Introduction

Bloodstream infection (BSI) caused by carbapenem-resistant *Klebsiella pneumoniae* (CRKP) is a major public health problem worldwide, which causes the multiple nosocomial outbreaks, high morbidity and high mortality [1, 2]. World Health Organization listed CRKP as one of the critical antibiotic-resistant bacterial pathogens in 2017, for which new antibiotics are urgently needed [3].

The mortality rates of patients with BSI caused by CRKP is reported to be significantly higher than that caused carbapenem-susceptible *K.pneumoniae* (CSKP) (30–50% versus 2.4–12.5%) [4, 5]. Identifying the risk factors for increased prevalence of CRKP BSI can help formulate effective intervention strategies to prevent outbreaks of CRKP BSI. A growing body of literature suggests that risk factors for CRKP infection include exposure to antibiotics, intensive care units (ICU), invasive devices, and intestinal colonization by *K. pneumoniae*(KP) [4, 6, 7]. The risk factors for fatal outcomes were confirmed to include mechanical ventilation, septic shock and CRKP isolation [4].

Population-based studies demonstrate that BSIs are more common in older people, due to a variety of factors such as comorbidity, immunocompromised, malnutrition, and environmental factors. More than 50% of the cases occurred in patients aged 65 and over [4, 5]. In a recent large series of assessments of community onset BSI in patients aged 65 and older, 37.5% of bacteremia was medically related [6]. Due to the high frequency of atypical clinical manifestations, the diagnosis of BSI in frail elderly remains a challenge [7]. In the absence of specific infection symptoms, senile symptoms such as weight loss, delirium, drowsiness, anorexia, fall and urinary incontinence may be the most important symptoms [8]. In two retrospective studies, the independent risk factors for elderly patients to obtain nosocomial BSI were age, bed rest status, the presence of intravascular access or gastrostomy at admission, and urinary incontinence [8, 9]. However, it is worth noting that, currently in China, there are few studies on the risk factors and mortality for elderly patients with bloodstream infection of CRKP*.*

The aim of this study was to assess risk factors associated with CRKP BSI and mortality among elderly patients in China, improving the awareness of medical institutions on the prevention and control of CRKP infections in the elderly.

## Methods

### Study design and patients

This study was conducted in the 2850 beds of First Affiliated Hospital of Sun Yat-sen University from January 2011 to December 2020. Patients aged ≥ 65 years with confirmed *K. pneumoniae* BSI were included. According to established criteria, *K. pneumoniae* BSI is defined as the presence of at least one positive blood culture with infectious symptoms and signs [5, 7]. *K. pneumoniae* BSI cases were identified from the microbiology laboratory database. Only the first episode of *K. pneumoniae* BSI was included. Patients with multibacterial BSI or patients with incomplete medical records were excluded. A total of 252 episodes were enrolled in this study.

Patients included were followed for 28 days from the day of the first positive blood culture. The CRKP was defined as an isolate with a minimum inhibitory concentration (MIC) of ≥ 2 μg/mL for ertapenem or ≥ 4 μg/mL for imipenem/meropenem, according to the CLSI criteria (Clinical and Laboratory Standards Institute, 2022). The outcome measured was death within 28 days of the first positive blood culture, respectively.

The data collected included information regarding demographics, underlying diseases, length of hospitalization, intensive care unit (ICU) admission, exposure to invasive procedure, antibiotic treatment, significant history of infection, immunosuppressive therapy in the 90 days prior to the date of BSI onset, microbiological data and patient outcomes.

### Microbiological methods

The strains were identified by the Vitek 2 system (bioMérieux, Marcy l’Etoile, France). The antibiotics susceptibility tests for the strains were examined by Gram-negative susceptibility (GNS) cards on the Vitek system (bioMérieux, Marcy l’Etoile, France). Susceptibility testing results were interpreted according to the criteria recommended by the Clinical and Laboratory Standards Institute (CLSI, 2022). *E. coli* ATCC 25,922 and *K. pneumoniae* ATCC 700,603 were used as the the quality control strains for susceptibility testing.

### Statistical analysis

Categorical variables are expressed in numbers and percentages. Continuous variables are showed as mean and standard deviation (SD) (normal distribution) or median and interquartile range (IQR) (non-normal distribution). Chi square test or Fisher’s exact test were used for comparing categorical variables. According to their distribution, Student’s test or Mann–Whitney U-test was analyzed for continuous variables. The results of univariate analysis were as follows: Odds ratio (or), 95% confidence interval (CI), and P value. Significant variables with *P* value of < 0.05 were then selected into binary logistic regression model for multivariate analysis to evaluate risk factors of CRKP BSIs. Risk factors for 28-day KP-BSI mortality were analyzed by cox logistic regression. Multivariable analysis including binary logistic regression and cox logistic regression using Forward LR method. Survival analysis was examined by Kaplan–Meier survival analysis.

During all the statistical analysis, variables with *P*-value < 0.05 were considered statistically significant. All statistical analyses were performed using IBM SPSS 24.0 software.

## Results

### Clinical and demographic characteristic of patients with KP BSI

Among these 252 KP BSI inpatients, 29 (11.5%) KP isolates were carbapenem-resistant. The overall 28-day mortality rate of elderly patients with a KP BSI episode was 10.7% (27/252). It was significantly higher for patients with CRKP BSI (14/29, 48.3%) than patients with CSKP BSI (13/223, 5.83%) (*P* < 0.001).

Most elderly patients (233/242, 92.5%) had at least one underlying disease, including hypertension (40.5%), previous bacterial infections (40.5%), solid organ tumors (37.3%), diabetes mellitus (27.0%), organ dysfunction (24.6%), septic shock (14.7%), hematologic malignancies (5.2%), and immunodeficiency (2.8%). Besides, 23.4% of these patients had been admitted to ICU department before BSI onset. The median duration of hospital stay before the onset of BSI was 6 days (IQR, 2 to 13 days). The clinical and demographic characteristics of cohort patients with KP BSI isolates were shown in Table 1.

**Table 1:** Characteristic of patients, univariate and multi-variate analysis of risk factors for BSI caused by CRKP compared with patients with BSI caused by CSKP

	Total (*n* = 252)	CSKP (*n* = 223)	CRKP (*n* = 29)	*P*	OR	*P*
Demographic variables						
Male sex	166(65.9)	143(64.1)	23(79.3)	0.105		
Age						
60–70	138(54.8)	123(55.2)	15(51.7)	0.78		
70–80	73(29.0)	65(29.2)	6(20.7)	0.66		
80–90	38(15.1)	33(14.8)	5(17.2)	0.44		
> 90	5(2.0)	2(0.9)	3(10.3)	0.26		
Co-morbidities						
Hypertension	102(40.5)	79(35.4)	23(79.3)	< 0.001	13.789(3.883–48.969)	< 0.001
Diabetes mellitus	68(27.0)	57(25.6)	11(37.9)	0.158		
Hematological tumors	13(5.2)	12(5.4)	1(3.5)	0.658		
Solid organ tumors	94(37.3)	86(38.6)	8(27.6)	0.25		
Previous bacterial infections	102(40.5)	85(38.1)	17(58.6)	0.034		
Septic Shock	37(14.7)	31(13.9)	6(20.7)	0.331		
Immunosuppression	7(2.8)	5(2.2)	2(6.9)	0.151		
Organ dysfunction	62(24.6)	54(24.2)	8(27.6)	0.692		
Antibiotics before KPN isolation						
β-Lactam/lactamase combinations	29(11.5)	21(9.4)	8(27.6)	0.009		
cephalosporins	47(18.6)	35(15.7)	12(41.4)	0.001		
carbapenems	45(17.9)	28(12.6)	17(58.6)	< 0.001	8.073(2.066–31.537)	0.003
quinolones	23(9.1)	19(8.5)	4(13.8)	0.354		
Hospital stays before onset	6(2,13)	5(1,12)	16(5,33)	0.016		
ICU stay	59(23.4)	38(17.0)	21(72.4)	< 0.001	11.180(2.663–46.933)	0.001
Pathogen isolated from other sites	96(38.1)	75(33.6)	21(72.4)	< 0.001		
Ward						
Internal medicine	128(50.8)	123(55.2)	5(17.2)	0.98		
Surgical	76(30.2)	73(32.7)	3(10.3)	0.98		
ICU	48(19.1)	27(12.1)	21(72.4)	< 0.001		
Invasive operation						
Surgery	68(27.0)	61(27.3)	7(24.1)	0.714		
Puncture	108(42.9)	90(40.4)	18(62.1)	0.026		
Catheter	28(11.1)	22(9.9)	6(20.7)	0.081		
Mechanical ventilation	31(12.3)	18(8.1)	13(44.8)	< 0.001		
Mortality	27(10.7)	13(5.8)	14(48.3)	< 0.001		

Data are expressed as n(%) or median(IQR)

### Risk factors for patients suffered from CRKP BSI versus CSKP BSI

The clinical characteristics of patients with CRKP BSIs and CSKP BSIs were compared in Table 1. The variables associated with CRKP BSI, using the univariate analysis, included hypertension, previous bacterial infections, and hospital stay before BSI onset. Further, ICU stay before BSI onset, department when KPN isolated, blood transfusion, mechanical ventilation, application of puncture, prior exposure to β-lactam/lactamase combinations, carbapenems, cephalosporins were identified associated with CRKP BSI.

Further, our multivariate analysis summarizes independent risk factors for developing CRKP BSI versus CSKP BSI: hypertension (OR: 13.789, 95% CI: 3.883–48.969, *P* < 0.001), exposure to carbapenems (OR: 8.073, 95% CI: 2.066–31.537, *P* = 0.003), and ICU stay (OR: 11.180, 95% CI: 2.663–46.933, *P* = 0.001).

### Risk factors for 28-day mortality in patients with KP BSI

The univariate analysis to identify potential risk factors for 28-day mortality of KP BSI include isolation of CRKP (OR 15.077, 95% CI 6.015–37.789, *P* < 0.001), combination with other site infection (OR 2.8, 95% CI 1.225–6.397), *P* < 0.001), sepsis shock (OR 4.314, 95% CI 1.792–10.385, *P* < 0.05), hospital stay (OR 1.023, 95% CI 1.009–1.036, *P* = 0.001), ICU stay before onset (OR 1.038, 95% CI 1.018–1.059, *P* < 0.001), infection with other pathogen(OR 7.000, 95% CI 2.711–18.076, *P* < 0.001), BSI onset in ICU (OR 25.667, 95% CI 9.514–69.241, *P* < 0.001), catheter (OR 5.552, 95% CI 2.155–14.300, *P* < 0.001), mechanical ventilation (OR 13.176, 95% CI 5.344–32.487, *P* < 0.001).

A multivariate analysis conducted on these 252 patients showed that variable associated with 28-day mortality in patients with KP BSI were CRKP isolation (OR 2.881, 95% CI 1.228–6.756, *P* = 0.015) and KP isolated in ICU (OR 11.731, 95% CI 4.226–32.563, *P* < 0.001) (Table 2).

**Table 2:** Univariate and multi-variate analysis of risk factors for 28-day mortality in patients with KP BSI

	Survival (*n* = 225)	Death (*n* = 27)	OR	*P*	OR	*P*
Demographic variables						
Male sex	145(64.4)	21(77.8)		0.167		
Age	69(64,76)	70(64,79)		0.134		
Carbapenem resistance	15(6.7)	14(51.9)	15.077(6.015–37.789)	< 0.001	2.881((1.228–6.756)	0.015
Co-morbidities						
Hypertension	87(38.7)	15(55.6)		0.091		
Diabetes mellitus	61(27.1)	7(25.9)		0.896		
Hematological tumors	12(5.3)	1(3.7)		0.718		
Solid organ tumors	85(37.8)	9(33.3)		0.652		
Infection(any)	85(37.8)	17(63.0)	2.8(1.225–6.397)	0.012		0.331
Septic Shock	27(12.0)	10(37.0)	4.314(1.792–10.385)	0.001		0.148
Immunosuppression	5(2.2)	2(7.4)		0.121		
Organ dysfunction	52(23.1)	10(37.0)		0.112		
Hospital stays before onset	5(1,12)	14(7,38)	1.023(1.009–1.036)	0.001		0.223
ICU stays before onset	0(0,1)	10(1,34)	1.038(1.018–1.059)	< 0.001		0.613
Pathogen isolated from other sites	75(33.3)	21(77.8)	7.000(2.711–18.076)	< 0.001		0.324
KP isolated in ICU	27(12.0)	21(77.8)	25.667(9.514–69.241)	< 0.001	11.731(4.226–32.563)	< 0.001
Invasive operation						
surgery	60(26.7)	8(29.6)		0.743		
puncture	23(10.2)	5(18.5)		0.195		
catheter	87(38.7)	21(77.8)	5.552(2.155–14.300)	< 0.001		0.060
Mechanical ventilation	17(7.6)	14(51.9)	13.176(5.344–32.487)	< 0.001		0.327
Appropriate empirical therapy	23(10.22)	1(3.7)		0.299		

Data are expressed as n (%) or median (IQR)

### Elder patients with KP BSI in ICU

**Fig. 1:**
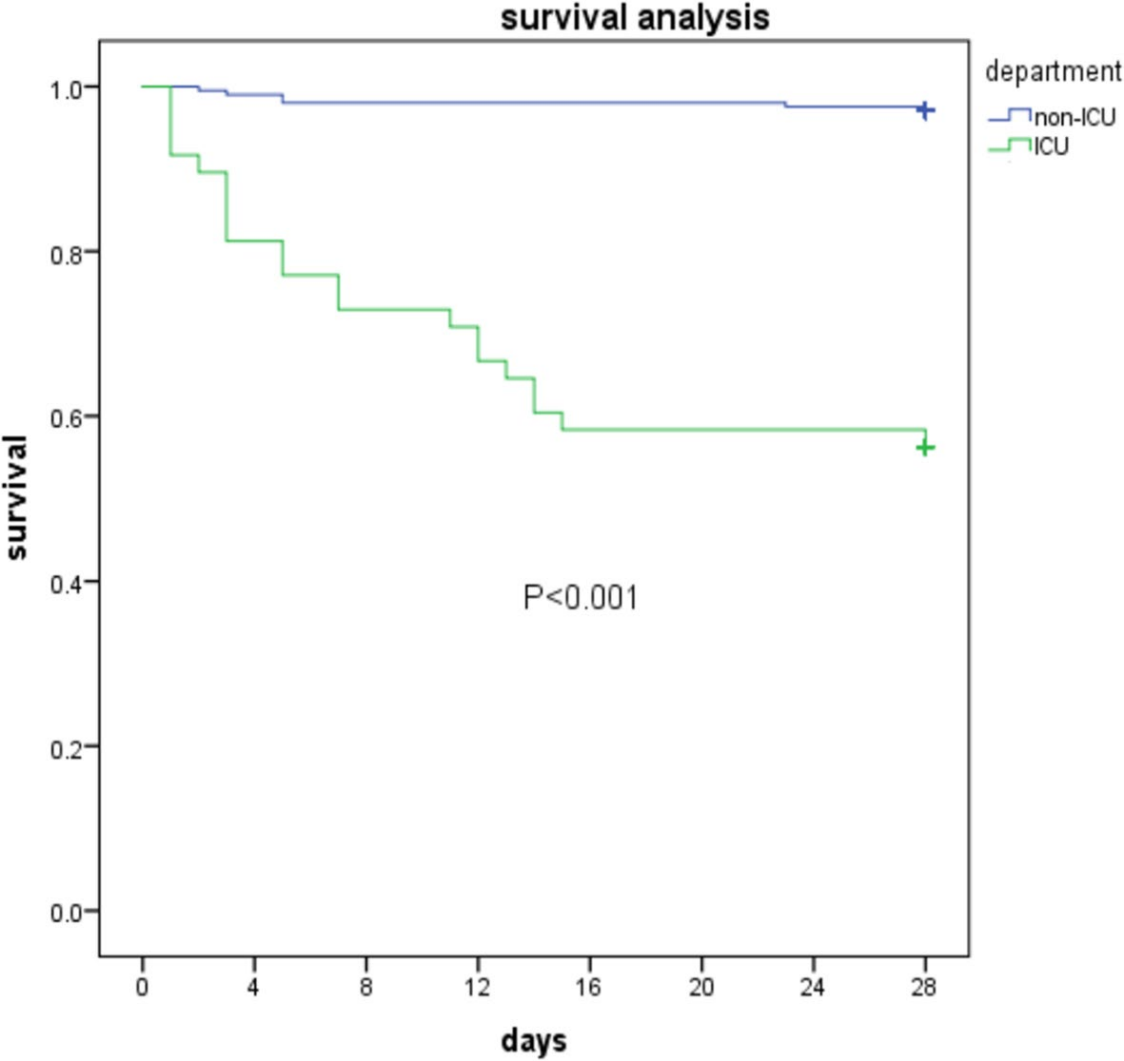
Kaplan–Meier curves of elderly patients in ICU (green line) and non-ICU (blue line)

ICU is a significant factor in patients which always means higher risk of carbapenem resistance and higher mortality. Kaplan–Meier survival analysis showed the significant of 28-days survival of patients in ICU versus non-ICU (*P* < 0.001) (Fig. 1).

**Fig. 2:**
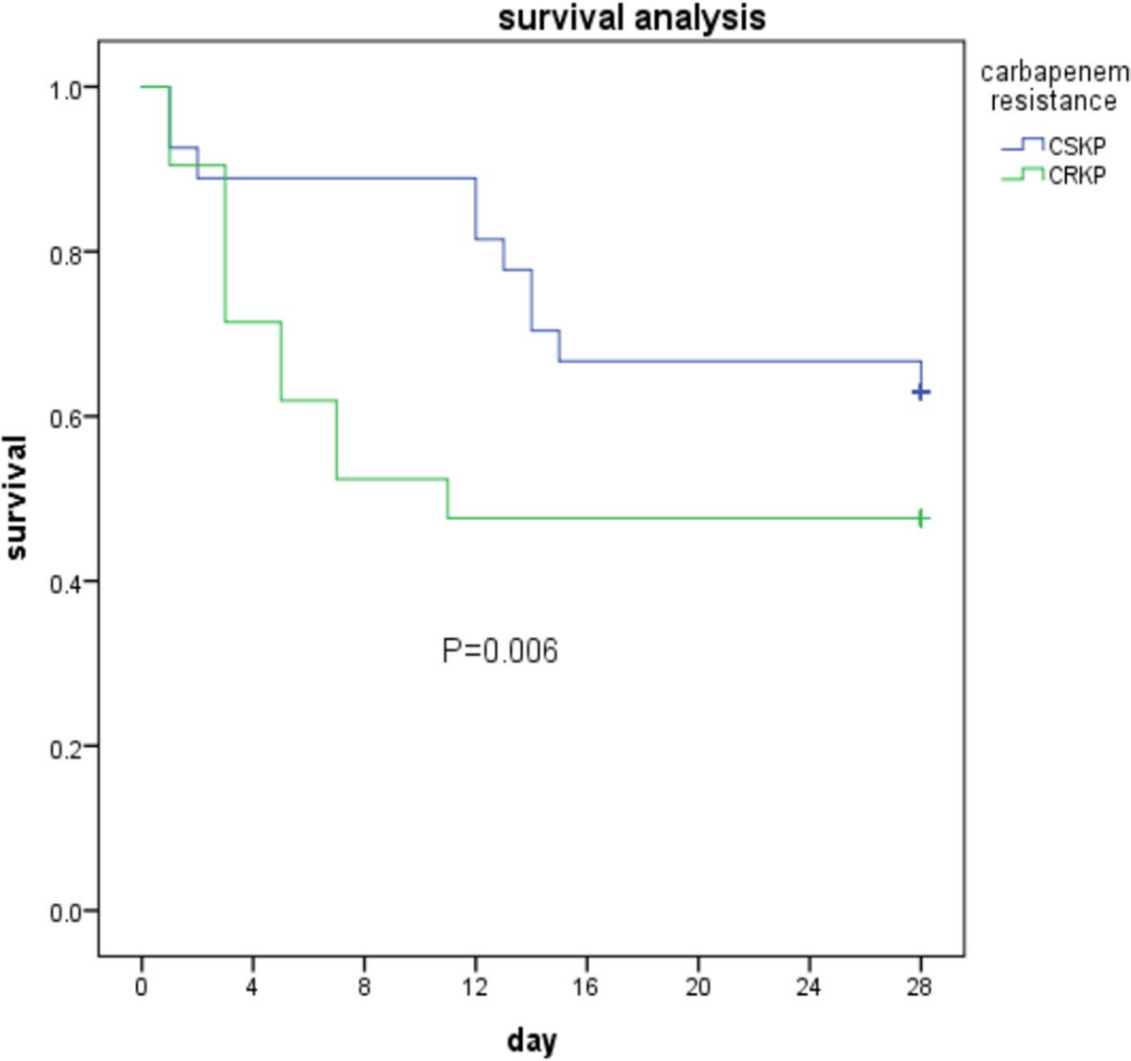
Kaplan–Meier analysis of elderly patients with CSKP-BSI and CRKP-BSI in ICU

Among 48 patients with KP BSI in ICU, the overall 28-day mortality rate of elderly patients with a KP BSI episode was 43.6% (21/48). It was significantly higher for patients with CRKP BSI (11/21, 52.4%) than patients with CSKP BSI (10/27, 37.0%) (*P* < 0.001) (Fig. 2). The median duration of ICU stay before the onset of BSI was 14 days (IQR, 1 to 33 days). Except patients have higher operation rate in SICU, there was no significant in characteristics between patients in MICU and SICU (Table 3).

**Table 3:** Characteristics of patients with KP BSI in ICU

	Total (*n* = 48)	MICU (*n* = 27)	SICU (*n* = 21)	*P*
Demographic variables				
Male sex	34(70.8)	18(66.7)	16(76.2)	0.471
Age	69(65–77)	69(65–77)	69(69–75)	0.834
Length of ICU stay	14(1,33)	5(1,31)	16(8,33)	0.184
Pathogen isolated from other parts	38(79.2)	24(88.9)	14(66.7)	0.06
surgery	13(27.1)	2(7.4)	11(52.4)	0.001
Carbapenem resistance	21(43.6)	15(55.6)	6(28.6)	0.062
Mortality	21(43.6)	15(55.6)	6(28.6)	0.062

Data are expressed as n(%) or median(IQR)

## Discussion

Population-based studies showed the incidence rate of BSI has been rising in recent years, especially in elderly patients. A recent study showed that the average incidence rate of age group over 65 was 6000 times per 100 thousand population [8, 9]. More than half of BSI occurs in patients aged 65 and over, and 70% of deaths occur in this age group, highlighting the severity of BSI in elderly patients [8]. *Klebsiella spp.*is the common pathogen causing BSI in the elderly, accounting for about 3%—10% [10]. It is worth noting that CRKP infection is becoming a serious problem and has attracted much attention due to limited treatment options and adverse effects on prognosis. However, currently, there is a lack of epidemiological data on BSI caused by CRKP in the elderly population, especially the studies on specific measures to prevent BSI infection of older patients are still insufficient.

In the present study, we described the clinical characteristics, risk factors and outcome of BSI due to KP in the elderly population, in order to reveal the severity of CRKP related bloodstream infection and provide a theoretical basis for further prevention of multidrug-resistant bacterial infection in the elderly patients. In this retrospective observational study with 252 elderly patients with KP BSI, we identified over 10% of the patients suffered BSI caused by CRKP. It is worth noting that the incidence of CRKP was the highest in the age group over 90 years old, suggesting that these patients should be vigilant about the isolation of CRKP in other parts to avoid further BSI. Almost all the study subjects have an underlying disease/comorbidity. The common underlying diseases/comorbidities of the study subjects included hypertension, previous bacterial infections, malignant tumors, diabetes mellitus, organ dysfunction and septic shock. This finding is similar to previous studies [11, 12]. It is reported that the most common source of BSI in older patients is the urinary tract, increasing with age, and accounting for 20–40% and up to 60% of bloodstream infections [13–15]. Compared with young patients, the elderly have a higher risk of BSI in pyelonephritis [14]. Our results showed that more than one fifth of patients had a history of admission to the ICU. Most of these patients were severe patients with consciousness disorder, limb movement disorder, language disorder and so on. They sufferd from a variety of underlying diseases, immunosuppression, exposure to a variety of antibiotics, and invasive operation, such as indwelling urinary catheter, gastric tube, endotracheal intubation, mechanical ventilation, etc., which increased the risk of bloodstream infection. Meanwhile, receipt of broad-spectrum antibiotics has also been identified as risk factors of CRKP BSI [5, 7]. The inadequacy of empirical antimicrobial regimens also emerged as a predictor of mortality of BSI caused by antibiotic resistant *Enterobacteriaceae* in the general populations. The present study proved that exposure to carbapenems was one of the independent risk factors for developing CRKP BSI in elderly.

In the present study, the 28 days-mortality of those who suffered from bloodstream infection caused by KP was 10.7% (27/252). This mortality was lower compared with another study with 46.2% (48/104) [16].The mortality associated with CRKP-BSI was significantly (48.3%) higher in elderly patients. Significantly, the mortality associated with CRKP-BSI in ICU patients was much higher. As identified in previous studies, ICU stay is a critical risk factor to develop CRKP BSI [17, 18]. According to a systematic review and meta-analysis, pooled mortality among 2462 patients infected with CRKP was 42.14%, while 21.16% in those infected with CSKP. The mortality of patients with bloodstream infection (BSI) was 54.30%, and 48.9% in patients admitted to the intensive care unit (ICU) [18]. In our study, totally 48 (48/252, 19.0%) patients developed KP-BSI in ICU and mortality of these patients was 43.6%, which was much higher than overall mortality (10.7%). It is worth noting that the separation rate of CRKP in ICU is significantly higher than that in ordinary ward. It can be detected in ICU environment and various equipment, including bed, table, floor and ventilators. In addition, patients admitted to the ICU are more likely to undergo invasive surgery, which will lead to a higher probability of CRKP-BSI. Studies have proved that KP colonization is another important risk factor for ICU infection [19, 20], and more than 50% of the infections are caused by the strains carried by themselves. More importantly, it is believed that the reason for obtaining CRKP BSI during ICU hospitalization may be that after the extensive use of broad-spectrum antibiotics, the pre-existing CRKP in the gastrointestinal tract is screened out to become dominant, which develops into sequent infections [21]. Therefore, screening for colonization on admission and intervention strategies are urgently needed in.

There were some limitations in this study. First of all, it was a retrospective study conducted in a single center, including 252 elderly patients. This may affect the ability to generalize the study results. Further large-scale prospective multicenter investigations are needed. Moreover, molecular characterization on the clinical isolates to examine the carbapenem resistance mechanisms was not performed in this study. To our knowledge, this is the first study in China to demonstrate the epidemiological characteristics of the risk factors and mortality of BSI caused by CRKP in the elderly for the last decade, which provides a useful basis for the diagnosis and treatment of KP BSI in the elderly.

In summary, hypertension, exposure to carbapenems and ICU stay were associated with the development of CRKP BSI in elderly patients. We also found a high mortality caused by *K. pneumoniae* BSI in elderly patients in ICUs. Active screening of CRE for high-risk groups, especially for elderly patients, is conducive to the early identification, treatment and control of CRE infections, so as to achieve the successful management.

## Data Availability

The datasets used and/or analyzed during the current study available from the corresponding author on reasonable request.

## Abbreviations

BSI: Bloodstream infection
CRKP: Carbapenem resistant Klebsiella pneumoniae
CSKP: Carbapenem susceptible Klebsiella pneumoniae
